# Daratumumab for the Treatment of Multiple Myeloma

**DOI:** 10.3389/fimmu.2018.01228

**Published:** 2018-06-04

**Authors:** Torben Plesner, Jakub Krejcik

**Affiliations:** ^1^Institute of Regional Health Science, University of Southern Denmark, Vejle Hospital, Vejle, Denmark; ^2^Department of Hematology, Vejle Hospital, Vejle, Denmark

**Keywords:** daratumumab, myeloma, CD38, immunomodulation, adenosine, complement, trogocytosis, neonatal Fc-receptors

## Abstract

This mini-review will summarize the present state of development of the CD38 antibody daratumumab for the treatment of multiple myeloma.

Now as we are close to the 10-year anniversary of dosing the first patient with daratumumab (March 26, 2008), it seems appropriate to review how far we have come in the development of this CD38 antibody for the treatment of multiple myeloma. Based on preclinical development by scientists at the Danish-Dutch biotech company Genmab in collaboration with scientists at the University Hospital in Utrecht, daratumumab was selected among several hundred CD38 antibodies for clinical development. It was clearly recognized at that time that there was an unmet need for new treatment options because of the poor prognosis of patients who were double refractory to both proteasome inhibitors and IMIDS. At the same time, there was a certain level of anxiety surrounding the clinical use of monoclonal antibodies because of a recent disaster with a CD28 antibody that had been tested in a clinical phase I trial the year before we started testing daratumumab. Also, the fact that the target molecule of daratumumab, CD38, is widely expressed in the human body was a cause of concern. In addition to being expressed by leukocytes, erythrocytes, platelets, and immature cells of the bone marrow, CD38 is also expressed by neuronal cells and glial cells of the central nervous system, peripheral nerves, pancreas islets cells, osteoclasts, skeletal muscle cells, cardiac muscle cells, and bronchial epithelium. Every precaution was taken during the initial phase I/II clinical trial GEN501 to avoid serious damage to occur to the patients because of unwanted reactions with normal tissues. The possibility of testing in animal models for toxicity of daratumumab was limited by the lack of cross reactivity of daratumumab with the CD38 molecule of other species. Six chimpanzees were chosen for preclinical testing of daratumumab, one of them died from a cytokine storm and others developed significant drops of the platelet counts. These adverse events have not been seen in humans.

The reason for moving forward with testing of daratumumab for the treatment of multiple myeloma was the very high level of expression of CD38 by myeloma cells. Preclinical studies showed that daratumumab may kill myeloma cells by complement-mediated cytotoxicity, by antibody-dependent cellular cytotoxicity, and by antibody-dependent cellular phagocytosis (1–3). Due to the anticipated risk of significant side effects, the initial clinical testing of daratumumab took offset from extremely low doses of antibody starting with 0.005 mg/kg with a step-by-step increase of the dose up to a planned maximum of 24 mg/kg (4). Because of the safety precautions during the trial it took 3 1/2 years to enroll the first 23 patients. When the dose of daratumumab had been increased to a level of 2 and 4 mg/kg, we started to see signs of clinical efficacy with a drop in the patient’s M-protein. This created a lot of interest and since at the same time we had seen no major side effects to the treatment subsequent enrollment into this and other clinical trials with daratumumab made rapid progress. Pharmacokinetic studies showed that target saturation may be achieved at a dose of 16 mg/kg with a schedule that was defined as eight weekly dosing, followed by eight bi-weekly dosing and then dosing of daratumumab every 4 weeks. A maximum tolerated dose was not reached at a dose up to 24 mg/kg. The superiority of 16 over 8 mg/kg dosing has been confirmed in a clinical trial (5).

The first clinical trials conducted with single agent daratumumab demonstrated that about 30% of patients with relapsed refractory myeloma may respond to daratumumab (6). Interestingly, about 50% of all the patients participating in the trials had a significant prolongation of survival although they did not fulfill the criteria for a response to daratumumab according to IMWG criteria. A plausible reason for this effect of daratumumab was revealed when studies conducted at the University hospital in Amsterdam in collaboration with Janssen demonstrated an immunomodulatory effect of daratumumab (7). Immunoregulatory cells belonging to the T cell, B cell, and monocyte–macrophage system express CD38 and are eliminated during treatment with daratumumab (Figure 1). Since these immunosuppressive cells may inhibit cytotoxic T cells from exerting antitumor control the elimination of the cells causes expansion of cytotoxic T cells in a clonal fashion in myeloma patients treated with daratumumab, a process that is correlated with the clinical response and most likely causally related to the improved survival seen even in patients who do not have a significant reduction in the M-protein. In addition to the immunomodulatory effect of daratumumab exerted *via* elimination of immuneregulatory cells, it was recently shown that antibody-mediated inhibition of the enzymatic activity of CD38 on cytotoxic T cells may directly boost the antitumor activity of these cells (8).

**Figure 1:**
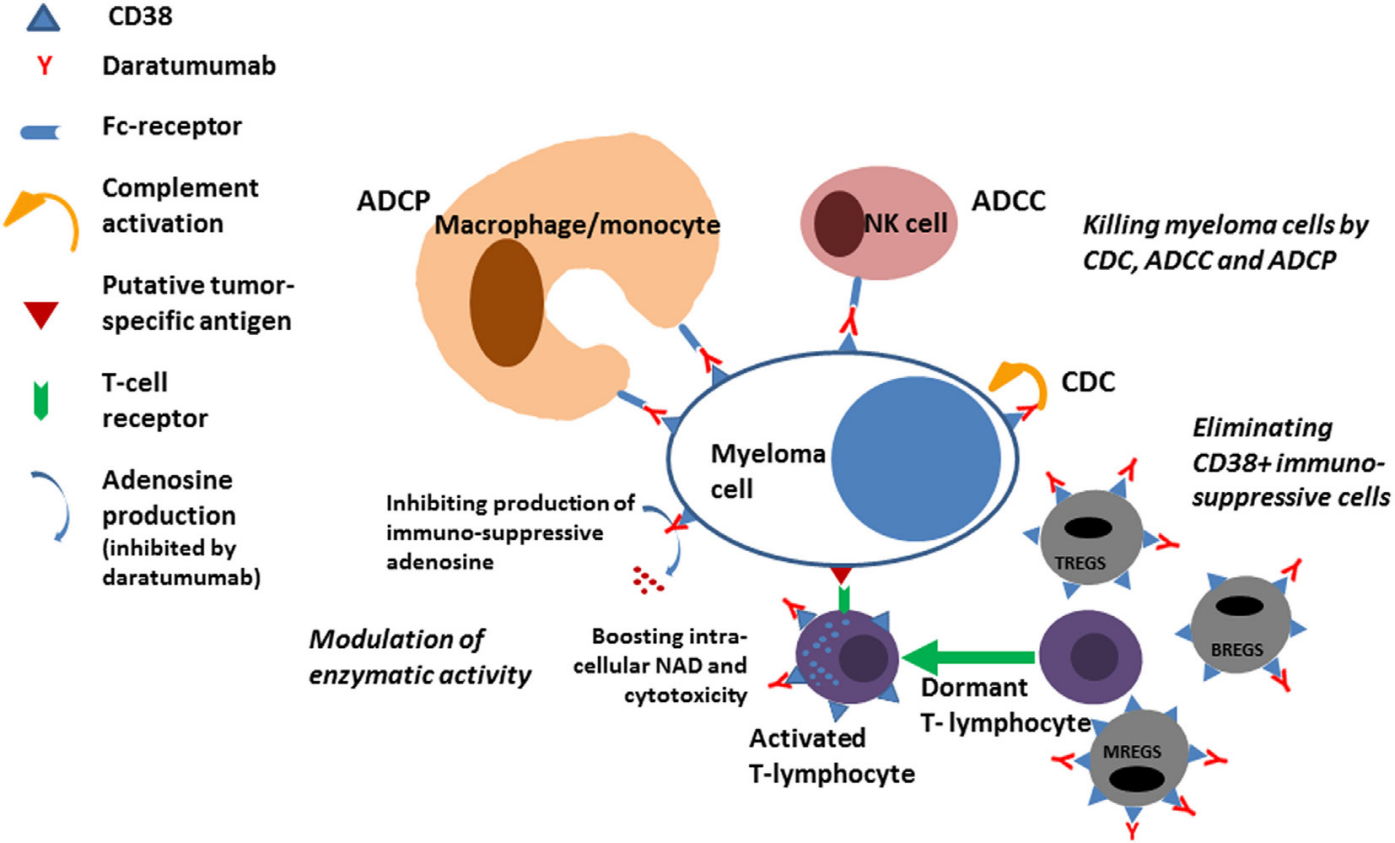
ADCP, antibody-dependent cellular phagocytosis; ADCC, antibody-dependent cellular cytotoxicity; CDC, complement-mediated cytotoxicity; TREGS, BREGS, and MREGS: regulatory cells of the T-cell, B-cell, and Myeloid-derived suppressor cells; NAD, nicotinamide adenine dinucleotide.

Preclinical studies of daratumumab *in vitro* and *in vivo* models had demonstrated significant additive or synergistic efficacy in combination with other anti-myeloma agents. These findings have now been confirmed in multiple clinical trials testing daratumumab in combination with many different anti-myeloma agents. The ability of daratumumab to combine with other anti-myeloma agents is excellent because there is no overlapping toxicity, and the impressive clinical response rates and duration of responses have now placed daratumumab in a very central position for the treatment of multiple myeloma in second and now also in first line (4–6, 9–14).

Still some patients fail to respond to daratumumab and some patients have progressive disease while being treated with daratumumab. The reason for failure of daratumumab is not understood. Immediately after initiating therapy with daratumumab, the level of CD38 expression by myeloma cells is reduced to much lower levels (15). However, this does not seem to be the cause of failure of the treatment because many patients continue to maintain a response despite the low levels of CD38 expression by myeloma cells. The reasons for low levels of CD38 expression by myeloma cells during treatment with daratumumab may be antibody-mediated “capping” of the daratumumab–CD38 complex on the plasma membrane followed by exocytosis or endocytosis and degradation of the antigen–antibody complex or due to rapid elimination of myeloma cells expressing high levels of CD38, or as recently shown due to “trogocytosis”, a process where phagocytes nipple fragments of the plasma membrane carrying antigen–antibody complexes (16).

At the time of failure of daratumumab, there is an increase in the expression of complement regulatory molecules such as CD55 and CD59 (15). These molecules may interfere with complement-mediated cytotoxicity and impair the clinical efficacy of daratumumab. It is also known that myeloma cells and cells in the microenvironment may express molecules such as PD-L1 that may interfere with the activity of cytotoxic T cells. Much hope has been put into combining daratumumab with checkpoint inhibitor antibodies such as PD-1 or PD-L1 antibodies to boost antitumor cytotoxicity. However, for the time being, the clinical trials in this field have been put to hold by FDA due to an excess mortality in the experimental arm of myeloma patients treated with checkpoint inhibitor antibodies and IMID in phase III trials.

The CD38 molecule is an ectoenzyme that may generate immunosuppressive adenosine and this process may be inhibited by daratumumab (17, 18). Thus, inhibiting the formation of immunosuppressive adenosine daratumumab may boost the T cell immune system and improve disease control. Immunosuppressive adenosine may be generated by CD38 expressed on the surface of myeloma cells, from CD38 expressed by cells in the microenvironment of the myeloma cells or, as recently suggested, by vesicles shed by myeloma cells and carrying CD38 out into the microenvironment surrounding the myeloma cells (19). Hypothetically, such microvesicles could, on top of contributing to generation of adenosine in the microenvironment, also cause off-target binding of daratumumab and contribute to treatment failure.

As it has been hypothesized that the low level of CD38 expressed by myeloma cells immediately after initiating treatment with daratumumab may be a reason for failure to respond to treatment attempts that have been made to increase the level of CD38 expression on myeloma cells with the hope to improve the efficiency of daratumumab (20). A clinical trial is now being conducted with ATRA in combination with daratumumab to increase CD38 expression by myeloma cells and improve responses. Preclinical studies have also shown that panobinostat may increase the expression of CD38 by myeloma cells and improve the response to daratumumab *in vitro* (21). However, our own limited clinical experience outside of a clinical trial testing panobinostat in combination with daratumumab for the treatment of patients progressing on daratumumab has not been successful.

In a model system of non-small cell lung cancer, it has been shown that CD38 is a growth and survival factor for the cancer cells (Gibbons D; ASCO-SICT Clinical Immuno-Oncology Symposium, February 23–25, 2017). Perhaps, the situation in myeloma is similar: high levels of CD38 may be beneficial for myeloma cell survival and conversely the low levels of expression imposed by treatment with daratumumab may render the myeloma cells more vulnerable to other anti-myeloma treatments. Recently, it was shown that myeloma patients refractory to daratumumab and lenalidomide when given separately may respond to the combination of daratumumab and lenalidomide (22). This could be due to daratumumab sensitizing myeloma cells to killing by lenalidomide or to boosting of an exhausted T-cell system in daratumumab refractory patients or both. The hypothesis that daratumumab may sensitize myeloma cells to other anti-myeloma agents fits well with the extraordinary good responses seen when daratumumab is combined with any other anti-myeloma agent not just IMIDs. If further substantiated that an implication may be that daratumumab should be part of any anti-myeloma treatment. Given the pleiotropic effects of daratumumab, it is in fact difficult to imagine how a myeloma patient can become truly refractory to daratumumab.

From a practical point of view, treatment with daratumumab is very easily managed, but it is important to take a few aspects into consideration.

The most important side effect is the infusion-related reaction that may occur during the first infusion in about half of the patients and rarely thereafter. It is a key to success to be prepared for this type of reaction, to look for subtle early signs of the reaction, and to pause the infusion and give extra premedication as soon as the first sign of an infusion-related reaction develops. Prior to the infusion, the patients receive pre-medications with glucocorticoids, antihistamine, montelukast, and paracetamol. If an infusion-related reaction develops the treatment with glucocorticoids and antihistamine can be repeated while the infusion is interrupted for about an hour. When the symptoms have subsided, the infusion can be resumed going back one step regarding the rate of infusion. Often the infusion can then gradually be accelerated and finished with only minor delay. It is recommended to give post-infusion medications with glucocorticoids for 2 days after the infusion, but we tend to admit that after the first two infusions of daratumumab to reduce the glucocorticoid exposure and risk of side effects. Patients with chronic obstructive pulmonary disease may require special attention and more prolonged treatment with glucocorticoids. From the third and subsequent infusions, the infusion rate of daratumumab can be increased so that the IV infusion is finished within 90 min (23). This is of importance in busy outpatient clinics where the number of patients in need of treatment often supersedes the space available. In future, the use of subcutaneous daratumumab that is now being developed in clinical trials may further improve the situation.

Another practical aspect to take into consideration is the expression of CD38 by erythrocytes. Consequently, immediately after starting treatment with daratumumab, this antibody will appear in the serum of patients as an irregular antibody that may cause trouble in the blood bank (24–27). To avoid delays in availability of blood units for transfusion, it is important to inform the staff at the blood bank about the situation, so they can be prepared and manage the difficulties. Daratumumab will cause reactivity in the *antibody screen test*, the *indirect Coombs test*, and the *crossmatch test* used by the blood bank to ensure that blood provided for transfusion will match the recipient. Although the erythrocytes express low levels of CD38, the direct Coombs test is not positive because erythrocytes binding daratumumab rapidly disappear from the circulation. Since only a very small drop of the hemoglobin level is observed after initiation of treatment with daratumumab, erythrocytes that have bound daratumumab may be cleared of the CD38–daratumumab complex on the plasma membrane by a process such as trogocytosis possibly mediated by phagocytes in the spleen and then recirculated.

The level and turnover of IgG in serum are regulated by the so-called “neonatal Fc-receptors” that may protect IgG from degradation. Thus, higher levels of serum IgG will tend to accelerate the turnover of IgG. In a recent study, it was found that despite identical dose levels and schedule the serum level of daratumumab is lower in patients with IgG versus patients with IgA myeloma (28). This did, however, not translate into a poorer response to daratumumab in patients with IgG myeloma. For myeloma patients receiving immunoglobulin replacement therapy to prevent infections, it may be advisable to separate as much as possible in time the infusion of daratumumab and normal human IgG, especially if the IgG is given intravenously resulting in high serum peak concentrations, since the amount of normal IgG infused is about 20 times higher than the standard dose of daratumumab.

Regarding assessment of response in myeloma, it is important to know that daratumumab may appear in the serum of patients as an IgG kappa-type M-protein. This may cause trouble when assessing the quality of a response to treatment, and therefore, new assays have been developed to discriminate between daratumumab and the patient’s own M-protein (29, 30). Often the level of M-protein represented by daratumumab is low, around 0.5–1 g/L, so when a patient approaches this level of M-protein it may be advisable to request the so-called “daratumumab interference assay” and then perform a bone marrow to confirm CR if it is shown that the residual M-protein is indeed daratumumab.

CD38 is a valuable marker for identification of plasma cells and together with CD138 it is routinely used to quantify the plasma cell compartment. However, during therapy with daratumumab a significant reduction of CD38 expression by MM cells occurs early during treatment (16). Therefore, other markers of plasma cells such as CD269 (BCMA) or CD319 (SLAMF7) may be needed as a substitute for CD38 (31, 32). In addition, daratumumab may affect the accessibility of some of the CD38 epitopes for binding of commercially available CD38 antibodies, so it is important to select an antibody which binds to an epitope on the CD38 molecule that is not occupied by daratumumab (33).

The efficacy and tolerability of daratumumab for the treatment of myeloma have led to rapid implementation of this new drug alone and in combination with standard of care anti-myeloma agents (Table 1). It has been approved by FDA, EMA, and many countries across the Globe for the treatment of relapsed-refractory myeloma, and data are now emerging from clinical trials that will likely result in the approval of daratumumab as first-line treatment of myeloma in combination with other drugs.

**Table 1:** Pivotal clinical trials evaluating daratumumab.

Study name (reference)	Number of patients	Patient population	Response	Adverse events
**GEN 501** Phase I/II daratumumab monotherapy (4)	32 patients in dose escalation 72 patients in cohort expansion	RRMM patients mostly refractory to bortezomib or lenalidomide with a median of four prior lines of treatment	16 mg/kg cohort ORR of 36%. 8 mg/kg cohort ORR of 10%. Median PFS in the 16 mg/kg cohort was 5.6 months. 65% of responding patients had not progressed after 12 months of follow-up.	No MTD in phase I (dose escalation up to 24 mg/kg) Daratumumab administration was safe with mostly mild grade 1–2 IRRs predominantly occurring during the first infusion. Most prominent IRRs were rhinitis, cough, or dyspnea.

**SIRIUS** Phase II daratumumab monotherapy (5)	124 patients 18 patients: 8 mg/kg 106 patients: 16 mg/kg	RRMM patients mostly refractory to bortezomib or lenalidomide with a median of five prior lines of treatment	16 mg/kg cohort ORR of 29% and median PFS was 3.7 months with a 1-year OS of 65%. 8 mg/kg cohort ORR 11%.	Similar to the GEN501 study, the most prominent side effects were IRRs of grade 1 or grade 2.

**GEN 503** Phase I/II daratumumab + lenalidomide + dexamethasone (13)	13 patients in dose escalation 32 patients in cohort expansion (16 mg/kg)	RRMM patients with a median of two prior lines of therapy	ORR was 84% in phase I and 81% in phase II with a total of 13 sCR, 3 CR, 13 VGPR, and 8 PR. In phase II, the 18-month PFS was 72% and OS was 90%.	Grade 3–4 adverse events (≥5%) included neutropenia, thrombocytopenia, and anemia. IRRs occurred in 56% of patients after the first infusion.

**POLLUX** Phase III Lenalidomide + dexamethasone **±** daratumumab (9)	569 patients. 286 in the DRd group and 283 in the control group	RRMM patients with a median of one prior line of therapy.	Higher ORR in the DRd group than in the control group (92.9% versus 76.4%). The hazard ratio for disease progression or death in the daratumumab group versus the control group was 0.37. In the DRd group the MRD-negative rate at 10^−5^ was 22.4% versus 4.6% in the Rd group.	Neutropenia grade 3 or 4 in 51.9% of the patients in the DRd group versus 37% in the control group. IRRs of grade 1 or 2 severity in 47.7% of the patients in the DRd group.

**CASTOR** Phase III bortezomib + dexamethasone **±** daratumumab (10)	498 patients. 251 in the DVd group and 247 in the control group	RRMM patients with a median of two prior lines of therapy.	Higher ORR in the DVd group than in the control group (82.9% versus 63.2%). The hazard ratio for disease progression or death in the DVd group versus the control group was 0.39. In the DVd group, the MRD-negative rate at 10^−5^ was 12 versus 2% in the control group.	|Thrombocytopenia grade 3 or 4 in 45.3% of the patients in the DVd group versus 32.9% in the control group. Neutropenia grade 3–4 was 12.8% for DVd versus 4.2% for Vd. |Daratumumab-related IRRs (mostly of grade 1 or 2) occurred in 45.3% of the patients, with the majority occurring during the first infusion.

**EQUULEUS** Phase Ib daratumumab + pomalidomide and dexamethasone (blood 130, Suppl. 1, 510)	103 patients	RRMM patients with a median of four prior lines of therapy.	ORR was 60%, median PFS 8.8 months and median OS 17.5 months. Out of 17 patients with CR or sCR, 29% were MRD negative by NGS at 10^−5^.	Neutropenia, anemia, fatigue, diarrhea, and thrombocytopenia were the most common side effects and considered to be mainly caused by pomalidomide. IRRs occurred in 50% of patients and mainly during the first infusion. One case of laryngeal edema occurred during the second infusion.

**ALCYONE** Phase III melphalan + prednisolon + bortezomib **±** daratumumab (11)	706 patients 350 in the D-MPV group and 356 in the control group	Newly diagnosed multiple myeloma patients who are ineligible for stem-cell transplantation.	ORR in the D-MPV group was 90.9 versus 73.9% in the control group. The hazard ratio for disease progression or death in the D-MPV group versus the control group was 0.5. In the D-MPV group, the MRD negative rate at 10^−5^ was 22.3 versus 6.2% in the MPV group.	Infections grade 3 or 4 in 23.1% of the patients in the D-MPV group versus 14.7% in the control group. Daratumumab-related IRRs (mostly of grade 1 or 2) occurred in 27.7% of the patients, with the majority during the first infusion.

*RRMM, relapsed/refractory multiple myeloma; ORR, overall response rate; PFS, progression-free survival; OS, overall survival; MTD, maximum tolerated dose; IRR, infusion-related reactions; MRD, minimal residual disease*.

## Author Contributions

Both the authors contributed to drafting and writing the manuscript.

## Conflict of Interest Statement

The authors declare that the research was conducted in the absence of any commercial or financial relationships that could be construed as a potential conflict of interest.
